# Vitamin C Prevents Hypogonadal Bone Loss

**DOI:** 10.1371/journal.pone.0047058

**Published:** 2012-10-08

**Authors:** Ling-Ling Zhu, Jay Cao, Merry Sun, Tony Yuen, Raymond Zhou, Jianhua Li, Yuanzhen Peng, Surinder S. Moonga, Lida Guo, Jeffrey I. Mechanick, Jameel Iqbal, Liu Peng, Harry C. Blair, Zhuan Bian, Mone Zaidi

**Affiliations:** 1 School of Stomatology, Wuhan University, Wuhan, Hubei, China; 2 The Mount Sinai Bone Program, Mount Sinai School of Medicine, New York, New York, United States of America; 3 Human Nutrition Research Center, Agricultural Research Service, United States Department of Agriculture, Grand Forks, North Dakota, United States of America; 4 Department of Pathology, University of Pittsburgh, Pittsburgh, Pennsylvania, United States of America; Oklahoma State University, United States of America

## Abstract

Epidemiologic studies correlate low vitamin C intake with bone loss. The genetic deletion of enzymes involved in *de novo* vitamin C synthesis in mice, likewise, causes severe osteoporosis. However, very few studies have evaluated a protective role of this dietary supplement on the skeleton. Here, we show that the ingestion of vitamin C prevents the low-turnover bone loss following ovariectomy in mice. We show that this prevention in areal bone mineral density and micro-CT parameters results from the stimulation of bone formation, demonstrable *in vivo* by histomorphometry, bone marker measurements, and quantitative PCR. Notably, the reductions in the bone formation rate, plasma osteocalcin levels, and *ex vivo* osteoblast gene expression 8 weeks post-ovariectomy are all returned to levels of sham-operated controls. The study establishes vitamin C as a skeletal anabolic agent.

## Introduction

The diligent search for small molecules and biologics to treat osteoporosis resonates with the expanding definition of osteoporosis and the implication that many more individuals worldwide have fragile bones. In developing nations in particular, while disease prevalence is difficult to estimate short of bone density measurements, the growing incidence of fractures poses a heavy burden of healthcare costs. In China, for example, almost 69 million individuals are estimated to have osteoporosis [1]. The cost of non-generic medications becomes difficult to bear in such emerging economies, prompting the need for affordable “osteoprotection”.

Vitamin C has long been known to affect the skeleton as gross deficiency causes the brittle bones of scurvy [2]. However, over the past decade, more subtle effects of vitamin C undernutrition have been gleaned. For example, low vitamin C intake is associated with low bone mass and a high fracture risk [3], [4]. More importantly, persuasive epidemiological evidence suggests that higher vitamin C intake is associated with higher bone mass [5], as well as reduced fracture risk over a 17-year follow-up [6]. Likewise, the Women's Health Initiative found a statistical relationship between total vitamin C intake and bone mineral density at both the hip and spine in women receiving hormone therapy [7]. Thus, it appears that, while adequate vitamin C prevents scurvy, higher doses might protect against skeletal loss.

Further evidence for an effect of vitamin C on bone mass comes from mouse genetic studies. The deletion of two key enzymes aldose reductase and aldehyde reductase, which results in absent *de novo* synthesis of ascorbic acid in mice, causes scorbutic bones [8]. While humans have lost the ability to synthesize vitamin C *in vivo*, and thus require nutritional supplementation, data in mice firmly establish an indispensible role for vitamin C in skeletal homeostasis. Both mouse and human osteoblasts require ascorbic acid to differentiate into mature mineralizing cells [9], [10]. In addition, mice that genetically lack ascorbic acid have immature dysplastic osteoblasts [8]. Thus, a key target for vitamin C appears to be the osteoblast. However, vitamin C also alters the resorption of bone by osteoclasts [11].

Importantly, Chambers and colleagues found that intraperitoneally injected ascorbic acid (2 mmol/kg/day) prevented ovariectomy-induced hyper-resorption and bone loss [12]. This study provided proof-of-concept that vitamin C could potentially be used to prevent hypogonadal bone loss. Still, even with the passage of ∼20 years, no clinical trials have evaluated the effect of vitamin C on skeletal integrity in humans. Here, we extend Chambers' initial observation, and provide evidence that vitamin C, when ingested orally, can prevent bone loss following ovariectomy through an anabolic action. We show, in a model of low-turnover osteoporosis, that this *in vivo* action results from the stimulation of bone formation noted in histomorphometric, bone marker, and quantitative PCR (qPCR) studies. Together with prior epidemiologic evidence showing a relationship between dietary intake and bone mass, our data provide compelling evidence for a therapeutic potential for vitamin C.

## Results

Groups of 6 month-old female C56/BL6 mice were ovariectomized or sham-operated and given vitamin C (5 mg/day) in drinking water *ad libitum*. Areal bone mineral density (BMD) was measured by *Piximus* (Lumar GE) at 0, 4 and 8 weeks, following which bones were harvested for micro-CT (Scanco μCT40) and histomorphometry measurements. Additionally, harvested bone marrow stromal cells were cultured in the absence of ascorbic acid for 6 and 10 days, following which qPCR was performed on the extracted RNA [13].

**Figure 1:**
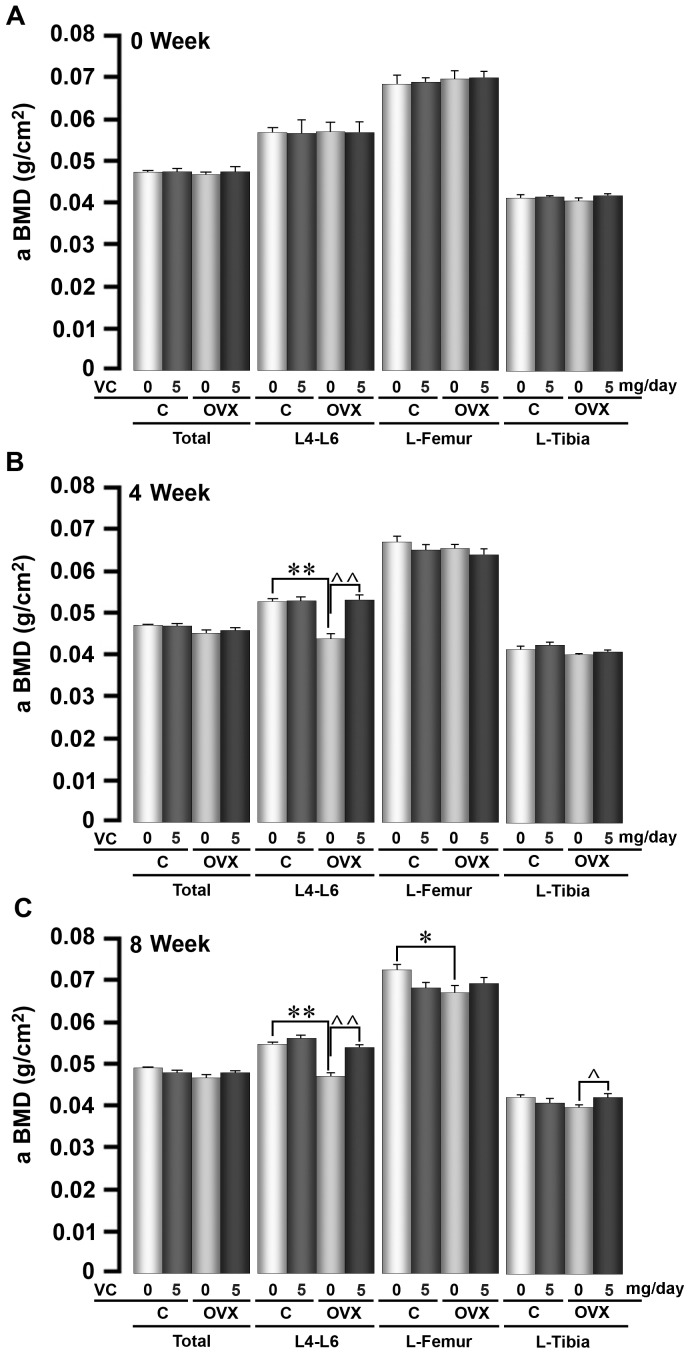
Oral Vitamin C Prevents Ovariectomy-Induced Bone Loss in Mice. Bone mineral density (BMD) measurements by *Piximus* (GE Lunar) at the total body, and the lumbar spine (L4–L6), left femur and tibia, measured 0, 4 and 8 weeks following ovariectomy (OVX) or sham operation (C) in 6 month-old mice. The mice were allowed to ingest vitamin C (VC) (5 mg/day) in drinking water *ad libitum*. Statistics: comparisons were made for differences between ovariectomized and sham-operated mice (*p<0.05, **p<0.01), and within the sham-operated and ovariectomized groups, between vitamin C treated and controls (∧p<0.05, ∧∧p<0.01); n = 5 mice per group.

At both 4 and 8 weeks of treatment, there was a significant decrease in areal BMD at the lumbar spine (L4–L6) in ovariectomized mice compared with sham-operated controls (Figures 1B and 1C). Whereas vitamin C did not elevate lumbar spine BMD in sham-operated mice, it prevented the lumbar spine BMD loss following ovariectomy (Figures 1B and 1C). Thus, there was a significant difference between BMD in ovariectomized mice and ovariectomized mice treated with vitamin C (Figures 1B and 1C). In contrast to the lumbar spine (L4–L6), which represents largely trabecular bone, BMD at cortical bone-rich sites, such as the femur, was reduced significantly only at 8 weeks post-ovariectomy (Figure 1C). This decline was prevented in vitamin C-treated ovariectomized mice, but the difference did not reach statistical significance (Figure 1C). In contrast, minimal, statistically insignificant, decrements in tibial BMD were noted at 8 weeks; this change was prevented with vitamin C so that there was a statistically significant difference between tibial BMD in ovariectomized mice and ovariectomized mice treated with vitamin C (Figure 1C). These data are consistent with the relatively minimal effects of ovariectomy on cortical bone over 8 weeks.

**Figure 2:**
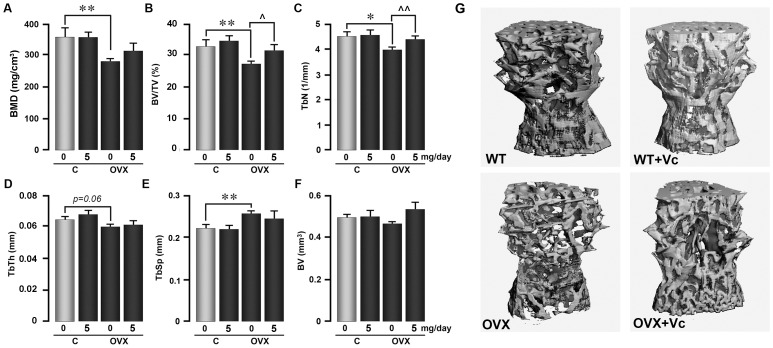
Oral Vitamin C Prevents Structural Deterioration in Ovariectomized Mice. Measurements by micro-CT (Scanco μCT40) of static parameters, including bone mineral density (volumetric) (A), bone volume fraction (BV/TV) (B), trabecular number (TbN) (C), trabecular thickness (TbTh) (D), trabecular spacing (TbS) (E), and bone volume (BV) (F), measured 8 weeks following ovariectomy (OVX) or sham operation (C) in 6 month-old mice. The mice were allowed to ingest vitamin C (VC) (5 mg/day) in drinking water *ad libitum*. Panel G shows representative μ-CT images from the respective groups. Statistics: comparisons were made for differences between ovariectomized and sham-operated mice (*p<0.05, **p<0.01), and within the sham-operated and ovariectomized groups, between vitamin C treated and controls (∧p<0.05, ∧∧p<0.01); n = 5 mice per group.

**Figure 3:**
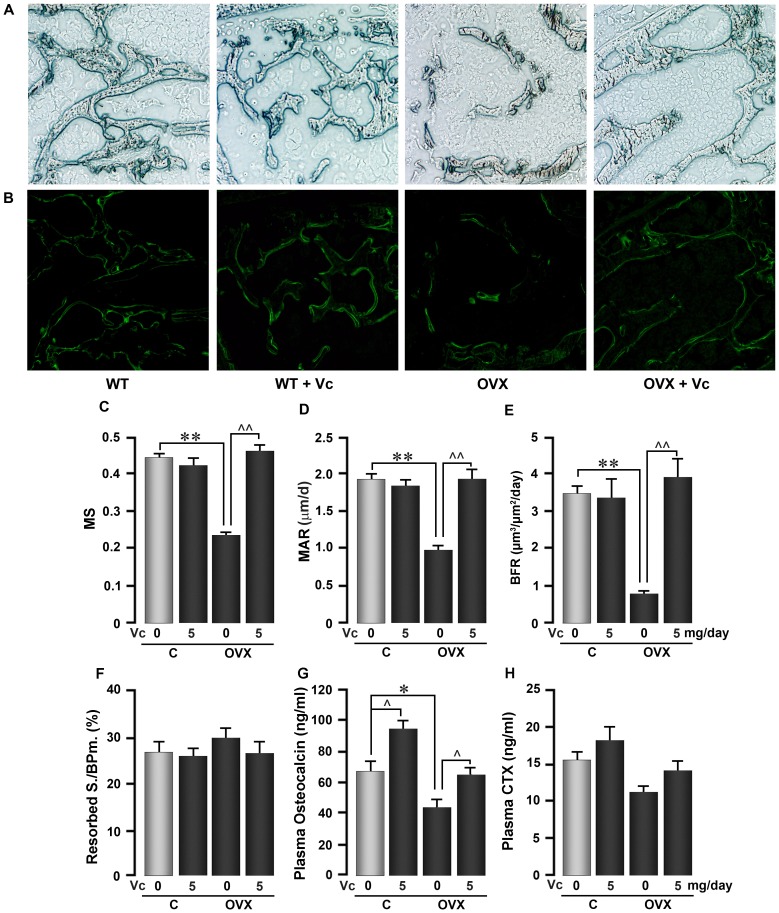
Oral Vitamin C Stimulates Bone Formation in Ovariectomized Mice. Representative reverse phase contrast (showing trabecular structure) (A) and fluorescence micrographs (showing calcein labels) (B) 8 weeks following ovariectomy (OVX) or sham operation (C) in 6 month-old mice. The mice were allowed to ingest vitamin C (VC) (5 mg/day) in drinking water *ad libitum*. Measurements of dynamic parameters, including mineralizing surface (MS) (C), mineral apposition rate (MAR) (D), bone formation rate (BFR) (E) and tartrate-resistant acid phosphatase- (TRAP-) labeled surfaces (Resorbed S./BPm) (F). Markers of bone turnover measured in plasma, namely osteocalcin (formation) (G) and C-telopeptide (resorption) (H). Statistics: comparisons were made for differences between ovariectomized and sham-operated mice (*p<0.05, **p<0.01), and within the sham-operated and ovariectomized groups, between vitamin C treated and controls (∧p<0.05, ∧∧p<0.01); n = 5 mice per group.

Micro CT analysis of the lumbar spine (L3) revealed that both BMD and BV/TV were reduced significantly upon ovariectomy (Figures 2A and 2B). However, while BV/TV prevented with vitamin C treatment, there was a statistically insignificant (p = 0.137) increase in volumetric BMD (Figures 2A and 2B). Likewise, trabecular number (Tb.N) was expectedly reduced and trabecular spacing (Tb.Sp) increased upon ovariectomy (Figure 2C and 2E). Only the decrement in Tb.N was, however, prevented with vitamin C treatment (Figure 2C). Figure 3A shows a two-dimensional view of the trabecular structure of bone that is dramatically reduced following ovariectomy and this effect was prevented with vitamin C treatment.

Figure 3 also shows data from dynamic histomorphometry of the lumbar spine. Calcein labeling was markedly reduced upon ovariectomy (Figure 3B), apparent as a profound reduction in mineralizing surface (MS), mineral apposition rate (MAR) and bone formation rate (BFR) (Figures 3C, 3D and 3E). No change in tartrate-resistant acid phosphatase (TRAP) labeled surfaces (Resorbed S./BPm) were noted when ovariectomized mice were compared with sham-operated controls (Figure 3F). The latter is not unexpected after 8 weeks of ovariectomy, when the initial hyper-resorption slows down and is accompanied by a decrease in bone formation – hallmarks of low-turnover osteoporosis [14]. Concordant results – notably a significant decline in plasma osteocalcin and a trend towards reduced plasma C-telopeptide – were noted upon ovariectomy (Figure 3G and 3H).

Vitamin C treatment completely prevented the reductions in MS, MAR and BFR to levels that were not significantly different from sham-operated controls (Figure 3C, 3D and 3E). This was paralleled by a similar reversal in plasma osteocalcin to control levels, with the difference that the treatment of sham-operated controls with vitamin C also significantly elevated plasma osteocalcin compared with the sham-operated controls (Figure 3G). Together, the data provide evidence for an anabolic action of vitamin C both in basal and ovariectomized states. No significant changes in resorption were noted upon vitamin C treatment, either in terms of resorbed S./BPm or plasma C-telopeptide levels (Figure 3F and 3H).

To obtain a long-term genetic imprint for the action of vitamin C, when administered *in vivo*, bone marrow stromal cells were cultured in the absence of ascorbic acid for 6 or 10 days. There were profound increases in the expression of the *bone sialoprotein protein* gene at both time points, whereas the early *bone morphogenetic protein-2* gene showed a significant increase at day 6, with minimal increases at day 10 (Table 1). While *Runx2* expression was unresponsive at day 6 of culture, vitamin C ingestion by sham-operated or ovariectomized mice triggered a significant increase in *Runx2* mRNA at day 10 (Table 1). Expression of the *osterix* and *alkaline phosphatase* genes were more sensitive to stimulation in sham-operated than in ovariectomized mice (Table 1); the reason for this difference is unclear. Despite the variations in the responsiveness of the respective genes to vitamin C, which may represent temporal expression differences, the data suggests that the vitamin C, administered *in vivo*, is a potent stimulator of osteoblast differentiation. The genetic imprint of this action is notable even after 10 days of *ex vivo* culture of stromal cells without added vitamin C.

**Table 1:** Oral Vitamin C Stimulates the Expression of Osteoblast Differentiation Genes.

	Day 6						Day 10					
	Sham			OVX			Sham			OVX		
VC	0	5		0	5		0	5		0	5	mg/day
***BSP***	1.00 ±0.10	5.05 ±0.37	**	1.00 ±0.08	4.57 ±0.37	**	1.00 ±0.07	16.8 ±1.14	**	1.00 ±0.87	3.86 ±0.32	**
***BMP2***	1.00 ±0.20	1.19 ±0.32		1.00 ±0.11	6.92 ±1.13	**	1.00 ±0.11	1.59 ±0.33	*	1.00 ±0.35	1.87 ±0.32	
***Runx2***	1.00 ±0.11	0.90 ±0.45		1.00 ±0.05	1.21 ±0.27		1.00 ±0.06	2.17 ±0.15	**	1.00 ±0.50	1.94 ±0.21	**
***Osterix***	1.00 ±0.18	1.47 ±0.17		1.00 ±0.52	0.83 ±0.06		1.00 ±0.08	3.81 ±0.11	**	1.00 ±0.50	1.63 ±0.08	**
***ALP***	1.00 ±0.06	2.68 ±1.01		1.00 ±0.06	0.85 ±0.07		1.00 ±0.06	4.24 ±0.78	**	1.00 ±0.39	1.42 ±0.49	

Quantitative PCR (qPCR) on RNA isolated from bone marrow stromal cells harvested from mice that were fed with vitamin C (5 mg/day, VC) following ovariectomy (OVX) or sham operation (Sham). The cells were cultured in ascorbate-free medium for 6 or 10 days following which the expression of several osteoblast differentiation genes and transcription factors, namely *bone sialoprotein* (*BSP*), *bone morphogenetic protein-2* (*BMP2*), *Runx2*, *osterix*, and *alkaline phosphatase* (*ALP*) were quantitated. Statistics: mean±SEM; comparisons between vitamin C-treated and untreated groups (*p<0.05, **p<0.01); qPCR in triplicate; n = 5 mice per group (pooled).

## Discussion

We examined the *in vivo* skeletal anabolic action of vitamin C in low-turnover osteoporosis induced 8 weeks following ovariectomy in mice [14]. In these mice, bone formation declined, whereas minimal changes were noted in resorption parameters. We show that vitamin C prevents bone loss in this model *via* a potent pro-osteoblastic action. This, however, does not rule out actions of vitamin C on bone resorption. A particular *caveat* is that earlier, strong transient increases in resorption post-ovariectomy have not been sampled in our study, while their inhibition may contribute to the effect of vitamin C on bone mass. Indeed, there is no doubt that vitamin C reduces bone resorption in high-turnover states, primarily by inhibiting osteoclastogenesis and promoting osteoclast apoptosis [11], [12], [15]–[17]. We have shown previously that the pro-osteoclastogenic effects of TNF-α are prevented by vitamin C [18]. Here, we show that ingested vitamin C additionally stimulates bone formation *in vivo*, likely by inducing the osteoblast to differentiate into a mature, mineralizing phenotype.

Our data complement the elegant loss-of-function studies by Gabbay and coworkers [8], in which they document a role for endogenous ascorbic acid on bone mass and bone remodeling in mice. They find that genetically deleting aldehyde reductase, an enzyme responsible for ∼85% *de novo* ascorbic acid synthesis, results in profound osteopenia, mainly due to reduced bone formation [8]. They identify dysplastic proliferating mesenchymal cell masses in nasal turbinates, reminiscent of similar masses seen the GLUO-deficient, scorbutic mouse [19]. They also note that castration further lowers bone mass, and is accompanied by severe osteoclastic bone resorption, which is rescued by feeding mice with ascorbic acid (1% chow pellets). As noted above, our complementary gain-of-function studies using a mouse model of low-turnover osteoporosis does not allow us to pin point the anti-resorptive actions of vitamin C. Instead, they reinforce the concept that ascorbic acid can also prevent bone loss through an anabolic action. Such anabolic actions have long been predicted from *in vitro* studies, towards which several molecular mechanisms have been proposed [9], [10], [20], [21]. Most notably, vitamin C is known to function as a co-factor for lysine and proline hydroxylation during type 1 collagen synthesis [22].

The ingested dose of vitamin C in our studies is 10-fold higher than over-the-counter doses (1000 mg) used as dietary supplements in humans. However, as mice synthesize the human body weight equivalent of 10,000 mg per day [23], a normal human daily dose of 1000 mg was considered insufficient to elevate concentrations above basal circulating levels in mice. Thus, we utilized a higher dose than the normal human dose, noting that Chambers and co-workers used an even higher dose, ∼1.7-fold higher (2 mmol/kg/day) than ours, to prevent bone loss in high-turnover osteoporosis [12]. This dose caused a ∼30% increase in circulating vitamin C levels.

There are very few therapies that selectively target low-turnover bone loss, such as that occurring with age and after high-dose glucocorticoid therapy [24]. The dysfunction or premature demise of osteoblasts in these conditions is a hallmark, reflected clinically in low bone formation markers. While anti-resorptive agents, such as bisphosphonates, that target the osteoclast, do reduce fracture risk even in low-turnover states, there are practical limits to their efficacy [25]. Anabolic agents, such as recombinant human parathyroid hormone (PTH) and a future anti-sclerostin antibody, make good pathophysiologic sense for use in these conditions. Vitamin C, through a combination of its known anti-resorptive action and the anabolic effect shown herein, may have a more profound effect on the skeleton.

However, it is possible that higher doses of vitamin C will be utilized compared with those recommended by the Institute of Medicine [26] or those offered in over-the-counter supplements. As a water-soluble vitamin, excess vitamin C is excreted in urine, and cases of vitamin C toxicity are therefore rare. The tolerable upper limit of intake of vitamin C is 2000 mg per day [26]. Beyond this level, rare side effects include mild gastrointestinal symptoms, such as abdominal cramps. However, reports for increased risk of kidney stones and of cardiovascular disease in postmenopausal women with diabetes are conflicting and inconclusive. It would nonetheless be worthwhile examining lower doses for additive or synergistic effects, particularly as adjuncts to current anti-resorptive and/or anabolic therapies. This begs the need for future dose-ranging and safety studies on vitamin C in humans.

## Materials and Methods

All animal studies were approved by the Institutional Animal Care and Use Committee at Mount Sinai School of Medicine and were performed in accordance with the guidelines of the National Institutes of Health and Mount Sinai School of Medicine.

The dose of vitamin C in our studies was 10-fold higher than over-the-counter doses (1000 mg) used as dietary supplements. However, a daily dose of 1000 mg was considered insufficient as mice synthesize the human body weight equivalent of 10,000 mg per day [23]. Chambers and co-workers used an even higher dose, ∼1.7-fold higher (2 mmol/kg/day) than ours [12]. This dose caused a significant (p = 0.038, by ANOVA, n = 19) ∼30% increase in circulating vitamin C levels from a mean (±SEM, µg/mL) of 30.4 (±3.54) to 39.2 (±3.51) (VITAMIN C ELISA, Biotang, Boston, MA). This suggests that the oral vitamin C dose was not only optimal in producing a significant change in serum vitamin C levels, but also efficacious in preventing ovariectomy-induced bone loss.

Areal bone mineral density (BMD) measurements were performed on C57/BL6J mice (Jackson Laboratories, Bar Harbor, ME) using a small animal bone densitometer (*Piximus*, Lunar-GE) [27]. Whole body BMD (minus cranium), and region-specific measurements of the spine (L4–L6), left femur, and left tibia were made. The instrument was calibrated each time before use by employing a phantom *per* manufacturer's recommendation.

For micro-CT estimations, the L3 vertebra was scanned non-destructively by using a Scanco μCT scanner (μCT-40; Scanco Medical AG, Bassersdorf, Switzerland) at 12 µm isotropic voxel size, with X-ray source power of 55 kV and 145 µA, and integration time of 300 milliseconds. The trabecular microstructure of the entire secondary spongiosa of L3 between the cranial and the caudal area was evaluated. The scanned grey-scale images were processed by using a low-pass Gaussian filter (sigma = 0.8, support = 1) to remove noise, and a fixed threshold of 220 was used to extract the mineralized bone from soft tissue and the marrow phase. The reconstruction and 3D quantitative analyses were performed using software provided by Scanco. The same settings for scan and analysis were used for all samples. Trabecular bone parameters included volumetric bone mineral density (BMD), bone volume (BV), bone volume fraction (BV/TV), trabecular thickness (Tb.Th), trabecular number (Tb.N), and trabecular spacing (Tb.Sp) (units specified in Figure 2).

Bone formation and resorption rates were quantitated by dynamic histomorphometry following two sequential injections of calcein (15 mg/kg) five days apart before sacrifice. Parameters included mineralized surface (MS), mineral apposition rate (MAR), bone formation rate (BFR), and TRAP surfaces (Resorbed S./BPm) [27]. Bone turnover markers, namely osteocalcin and C-telopeptide, were measured on mouse plasma using commercial kits (Mouse Osteocalcin kit, Biomedical Technologies, Stoughton MA, BT-470 and RatLaps EIA, Immunodiagnostics Systems, AC-06F1, respectively). Bone marrow was isolated and stromal cell cultures performed in the absence of ascorbate for 6 or 10 days. RNA was extracted for quantitation of gene expression using established protocols [13]. Quantitative PCR was performed as described [13]. L-ascorbic acid was purchased from Sigma.

The data were analyzed using Student's t-test with Bonferroni's correction. In every case, comparisons have been made for differences between ovariectomized and sham-operated mice to examine the effect of ovariectomy. Furthermore, within the sham-operated and ovariectomized groups, comparisons were made for differences between vitamin C treated and control mice. Results were considered significant if p<0.05.
